# A new rhynchocephalian (Reptilia: Lepidosauria) from the Late Jurassic of Solnhofen (Germany) and the origin of the marine Pleurosauridae

**DOI:** 10.1098/rsos.170570

**Published:** 2017-11-08

**Authors:** Gabriel S. Bever, Mark A. Norell

**Affiliations:** 1 Center for Functional Anatomy and Evolution, Johns Hopkins University School of Medicine, Baltimore, MD 21205, USA; 2 Division of Paleontology, American Museum of Natural History, Central Park West at 79th Street, New York, NY 10024, USA

**Keywords:** Bavaria, marine reptile, secondarily aquatic, skeletal development, sphenodontia, tethys

## Abstract

A new rhynchocephalian is described based on a recently discovered and well-preserved specimen from the Late Jurassic (Kimmeridgian) marine limestones of Solnhofen, Bavaria. Phylogenetic analysis recovers the new taxon as the sister group to Pleurosauridae, a small radiation of rhynchocephalians representing the oldest marine invasion of crown-clade Lepidosauria. The relatively strong evidence for this taxonomically exclusive lineage, within a generally volatile rhynchocephalian tree, places the new taxon in a position to inform the early history of the pleurosaur transition to the sea. The early steps in this transition are distributed throughout the skeleton and appear to increase hydrodynamic efficiency for both swimming and aquatic feeding. This early history may also have included a global truncation of plesiomorphic ontogenetic trajectories that left a number of skeletal features with reduced levels of ossification/fusion. The exact degree to which *Vadasaurus* had adopted an aquatic ecology remains unclear, but the insight it provides into the origin of the enigmatic pleurosaurs exemplifies the potential of Rhynchocephalia for generating and informing broad-based questions regarding the interplay of development, morphology, ecology and macroevolutionary patterns.

## 1. Introduction

Rhynchocephalia is a lineage of lepidosauromorph reptiles whose humble contribution to the extant vertebrate fauna is a single species, *Sphenodon punctatus*, surviving on 32 islands off the coast of New Zealand [1–4]. These populations belie a surprisingly deep evolutionary history that begins approximately 240 Ma and includes more than 40 known fossil taxa [2,5,6]. The structural diversity now recognized in the rhynchocephalian fossil record calls into serious question the notion that a want of evolvability rendered Rhynchocephalia incapable of outcompeting true lizards (Squamata) and/or mammals for control of overlapping terrestrial niche space (see [2,7–10]). Numerous examples can now be offered as evidence against this general hypothesis (see [8])—from a large-bodied herbivore that walked on hoof-like unguals (*Priosphenodon* [11]) to a running form with elongate hindlimbs (*Homeosaurus* [12]) to an armoured species covered in a complex network of dermal osteoderms (*Pamizinsaurus* [13]). One of the more compelling demonstrations of the evolutionary potential of the rhynchocephalian body plan, however, is found in the pleurosaurs, who left behind their ancestral terrestrial ecology for life in the water.

Pleurosauridae is a small rhynchocephalian clade consisting of only three recognized species: *Pleurosaurus goldfussi* [14], *Pleurosaurus ginsburgi* [15] and *Palaeopleurosaurus posidoniae* [16]. All three species are based on specimens collected from the Jurassic-aged marine limestones (Plattenkalk) of southern Germany and southeastern France. *Palaeopleurosaurus* is recovered from beds approximately 30 Myr older than those producing *Pleurosaurus* (lower Toarcian versus lower Tithonian). All three species express derived features widely interpreted as adaptations for an aquatic ecology. There is marked elongation of the trunk, tail and skull, producing a slender eel- or snake-like body form capable of at least somewhat efficient anguilliform (axial undulatory) swimming [16–18]. The girdles and limbs, which probably were used more for steering than propulsion in the shallow marine waters, exhibit marked reduction (especially the forelimbs). There is a general reduction of ossification levels and skeletal fusion, with one exception being the loss of caudal autotomy [16,17,19,20]. The terrestrial-to-marine transition evidenced by the pleurosaurs represents the stratigraphically earliest such invasion for crown-group Lepidosauria—a clade that would later produce a number of aquatic and semi-aquatic lineages including the fully marine mosasaurs (Mosasauridae) [21] and sea snakes (Hydrophiinae) [22] (all from the squamate side of the lepidosaur radiation).

The aquatic adaptations of *Palaeopleurosaurus* are not as pronounced as those of the stratigraphically higher *Pleurosaurus.* Its trunk and tail, while still derived, are less elongate than those of *Pleurosaurus*, its limbs are not as short, and its skull shape is less stretched and not as dorsoventrally flattened or triangular in the dorsoventral view [7,16,17,20]. The intermediate quality of these features cast *Palaeopleurosaurus* as an important transitional form between what we can safely infer was a fully terrestrial pleurosaur ancestor and the marine-adapted species of *Pleurosaurus*. The earliest history of this transformation series, basal to *Palaeopleurosaurus*, remains elusive. This limits our ability to infer the relative timing of synapomorphy accrual within this transition and thus the tempo and mode of its evolution. The especially unstable nature of the more deeply nested lineages within the rhynchocephalian tree [23,24] further complicates the situation by obfuscating the identity of the pleurosaur sister group and thus the morphological, ecological, stratigraphic and geographical details of pleurosaur origins. These difficulties emphasize the need for new specimens that inform this early history.

Here, we describe a new rhynchocephalian based on a well-preserved specimen from the Late Jurassic marine limestones of Solnhofen, Bavaria. Current understanding of rhynchocephalian skeletal variation supports the new taxon as the sister to Pleurosauridae. The new taxon thus provides valuable insight into the morphological details of pleurosaur origins, including the possibility that this secondarily aquatic group evolved as part of a radiation of Jurassic rhynchocephalians endemic to the European Archipelago of the northern Tethys Sea.

## 2. Material and methods

The cranial end of the specimen, including the skull and anteriormost vertebrae, was μCT scanned at the American Museum of Natural History Microscopy and Imaging Facility (AMNHMIF) using a copper filter, an air wedge, a voltage of 150 kV and a current of 124 mA. The constraints of mounting the entire slab for scanning necessitated that the specimen was scanned at an angle oblique to the standard coronal axis. A total of 534 slices were acquired, each with a resolution of 1024 × 1024 pixels in a reconstructed field of view of 35 mm. The voxel size (millimetre) is 0.03403 × 0.03403 × 0.03403. The geometry of slab specimens is not conducive to acquiring high-resolution images with a clear distinction between bone and matrix [25,26], and this certainly was the case with AMNH FARB 32768. Even with the overall poor quality of the CT images, we were able to distinguish several features unobservable in the external view. Some digital segmentation was attempted using Amira 6.0, but few cranial structures could be confidently isolated in their entirety. Our description focuses on the most phylogenetically relevant features. A more comprehensive description and comparison will be published elsewhere.

The phylogenetic relationships of AMNH FARB 32768 were assessed using maximum-parsimony (TNT 1.1; [27]) and a matrix of 33 taxa (31 rhynchocephalians) and 87 skeletal characters (electronic supplementary material, S1). Heuristic searches were conducted using tree bisection–reconstruction branch swapping with 1000 replicates of random stepwise sequence addition, and following recent rhynchocephalian studies, *Youngina capensis* was specified as the outgroup. Minimum branch lengths were set to collapse. Support for each node was measured by calculating Bremer support and bootstrap frequencies, with 1000 bootstrap replicates and 1000 random addition replicates. The matrix was compiled largely from previous studies [11,17,28–30]. Nineteen characters were recognized as ordered morphoclines (electronic supplementary material, S1). The phylogenetic implications of this approach were assessed by comparing the ordered results with those of iterations where all, or a subset, of characters were analysed unordered. A second set of parsimony analyses were run using a taxonomically restricted sample that excluded nine taxa known only from highly incomplete material (such as lower jaws). These taxa include *Cynosphenodon huizachalensis* [31], *Godavarisaurus lateefi* and *Rebbanosaurus jaini* [32], *Kaikaifilusaurus calvo* [33], *Kawasphenodon expectatus* [34], *Sphenocondor gracilis* [28], *Sphenovipera jimmysjoi* [24], *Theretairus antiquus* [35] and *Toxolophosaurus cloudi* (see [36]).

Bayesian estimations of phylogeny were calculated to assess the posterior probabilities of the various clades. The Mk model [37] was employed in MrBayes (v. 3.2.2) to analyse the taxonomically inclusive matrix with γ-distributed rate variation and variable coding. All analyses were performed with a sampling frequency of 1000, two concurrent runs and four Metropolis-coupled chains (*T* = 0.1) for 10 000 000 generations. The same 19 characters were treated as ordered. The analyses were checked for convergence using standard MrBayes diagnostics (for example, PRSF less than 0.01, mixing between chains greater than 20%) and Tracer (v. 1.5) (for example, ESS > 200). A 25% relative burn-in was implemented for all summary statistics.

## 3. Systematic palaeontology

Lepidosauria Haekel, 1866 (*sensu* [38])

Rhynchocephalia Günther, 1867 (*sensu* [39])

*Vadasaurus herzogi* gen. et sp. no.

### 3.1. Etymology

Generic name from the Latin *vadare* ‘to go forth’, which is also the root of ‘to wade’—refers to the taxon's hypothesized phylogenetic position near the proximal end of a terrestrial-to-marine transformation series that produced the aquatic pleurosaurs—and *saurus ‘*lizard’*.* The specific epithet honours the celebrated Bavarian film-maker Werner Herzog for his continuing exploration of the relationship between life and time.

### 3.2. Holotype

**Figure 1.**
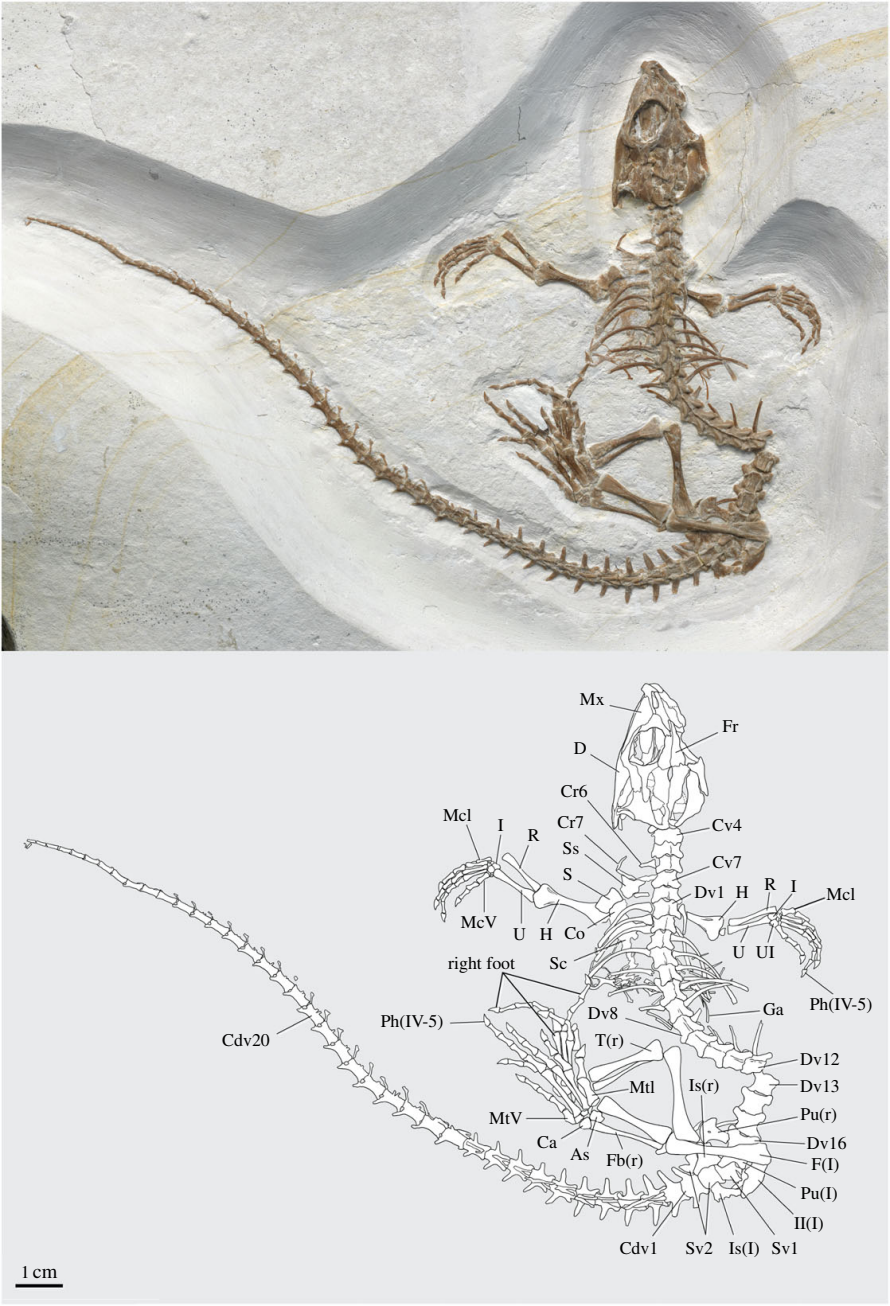
Holotype of *Vadasaurus herzogi* (AMNH FARB 32768) collected from the Late Jurassic marine limestones of Solnhofen, Bavaria. The skull, forelimbs, and first 18 presacral vertebrae and ribs are exposed in the dorsal or dorsolateral view. Posteriorly, the skeleton is rotated approximately 180°, making it visible largely in the ventral view. Left hindlimb is exposed in the dorsal view. Anatomical abbreviations: As, astragalus; Ca, calcaneum; Cdv, caudal vertebra; Co, coracoid; Cr, cervical rib; Cv, cervical vertebra; D, dentary; Dv, dorsal vertebra; F, femur; Fb, fibula; Fr, frontal; Ga, gastralia; H, humerus; I, intermedium; Is, ischium; l, left; Mc, metacarpal; Mt, metatarsal; Mx, maxilla; Ph, phalanx; Pu, pubis; R, radius; r, right; S, scapula; Sc, sternal cartilage; Ss, suprascapular cartilage; Sv, sacral vertebra; T, tibia; U, ulna.

**Figure 2.**
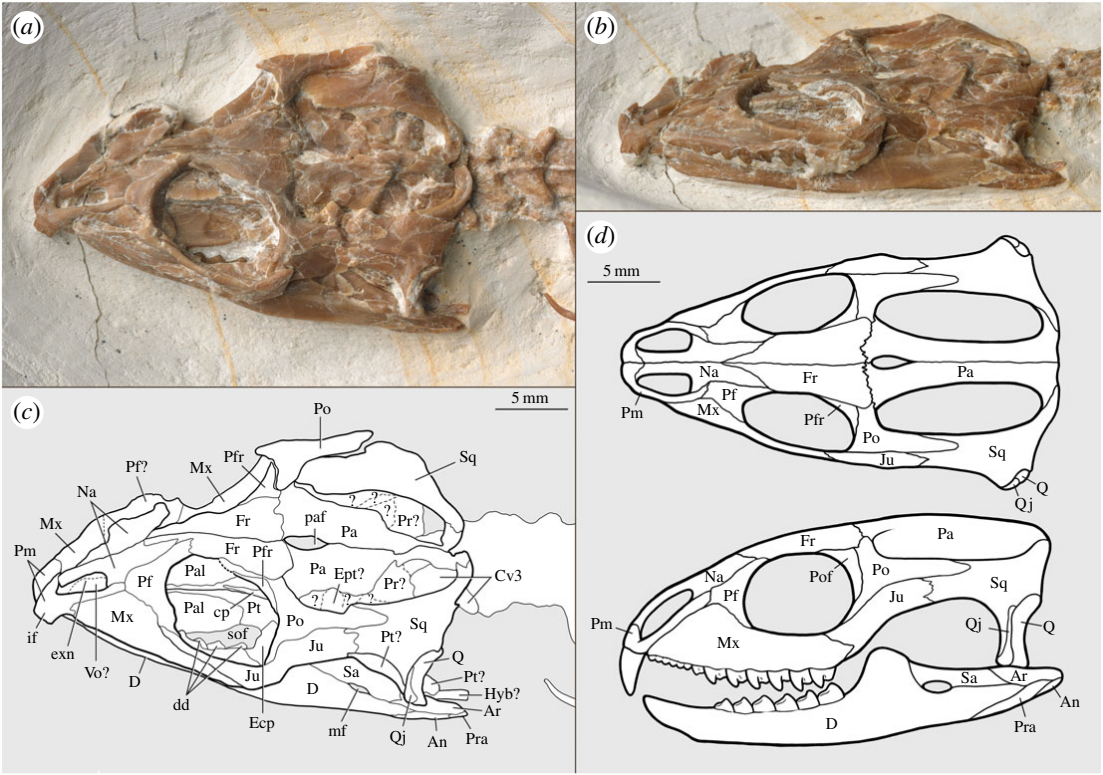
The skull of *Vadasaurus herzogi* (AMNH FARB 32768). Photographs in the dorsolateral (*a*) and lateral (*b*) views; labelled line drawing in the dorsolateral view (*c*); reconstructions of lateral and dorsal views (*d*). Anatomical abbreviations: An, angular; Ar, articular; cp, cultriform process; Cv, cervical vertebra; D, dentary; dd, dentary dentition; Ecp, ectopterygoid; Ept, epipterygoid; exn, external naris; Fr, frontal; Hy, hyobranchial element; if, incisiform fang; Ju, jugal; mf, mandibular foramen; Mx, maxilla; Na, nasal; Pa, parietal; Pal, palatine; paf, parietal foramen; Pf, prefrontal; Pm, premaxilla; Po, postorbital; Pof, postfrontal; Pr, prootic; Pra, prearticular; Pt, pterygoid; Q, quadrate; Qj, quadratojugal; Sa, surangular; sof, suborbital fenestra; Sq, squamosal; Vo, vomer.

**Figure 3.**
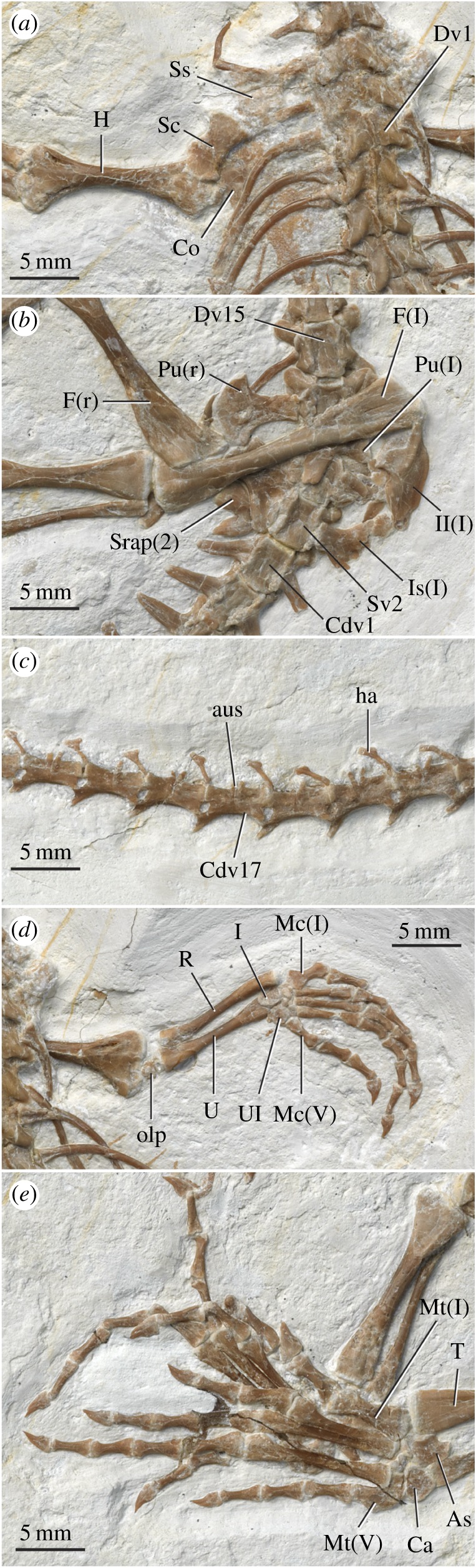
Selected photographs of AMNH FARB 32768 showing close-ups of the left shoulder girdle and surrounding elements (*a*), pelvis and surrounding elements (*b*), middle portion of the caudal vertebral series (*c*), right forelimb (*d*), and left foot (*e*). Anatomical abbreviations: As, astragalus; aus, autotomic septum; Ca, calcaneum; Cdv1, first caudal vertebra; Co, coracoid; Dv1, first dorsal vertebra; Dv15, fifteenth dorsal vertebra; F, femur; H, humerus; ha, haemal arch; I, intermedium; Il, ilium; Is, ischium; l, left; Mc(I), metacarpal one; Mc(V), metacarpal five; Mt(I), metatarsal one; Mt(V), metatarsal V; olp, olecranon process; Pu, pubis; R, radius; r, right; Sc, scapula; srap(2), rib of the second sacral vertebra; Ss, suprascapular cartilage; Sv(2), second sacral vertebra; T, tibia; U, ulna, Ul, ulnare.

AMNH FARB 32768, a nearly complete and largely articulated skeleton (figures 1–3). Like most specimens preserved in lithographic limestone, it exhibits compressional effects that include the flattening and shearing of composite structures and the slight displacement of certain elements. Individual bones, however, are preserved largely in three dimensions.

### 3.3. Type locality and age

Late Jurassic, uppermost Kimmeridgian, *Hybonotum* zone, *Ulmense* subzone, *Rebouletianum* horizon.

### 3.4. Diagnosis

AMNH FARB 32768 is diagnosed as a new rhynchocephalian taxon by a derived combination of the following features: elongate process of the premaxilla, which contacts the prefrontal thereby excluding the maxilla from the external nares, a short nasal process of the premaxilla, postfrontal participation in the upper temporal fenestra, expanded lower temporal fenestra constituting more than a quarter of the total skull length, loss of the subtemporal process of the jugal, elongate dorsal process of the jugal, ascending process of the quadratojugal reaching the midpoint of the temporal plate, low and rectangular neural arches, no distal contact between sacral ribs, robust metatarsals I and V, and more than 40 caudal vertebrae.

Diagnosed as a rhynchocephalian based on an elongate, posterior process of the dentary that reaches the glenoid cavity, acrodont marginal teeth bearing prominent flanges, marginal teeth regionalized with juvenile dentition positioned anteriorly, premaxillary teeth reduced to three, anterior contact of contralateral pterygoids, hourglass-shaped dorsal centra (in the ventral view), prominent second sacral vertebra with bifurcated sacral rib, first caudal vertebra lacking a haemal arch and a large posterior process of the ischium. Diagnosed as deeply nested within Rhynchocephalia based on the absence of individuated lacrimal, fusion of the premaxillary teeth into a chisel-like fang, square-shaped marginal teeth with a prominent flange, no more than two successional teeth, edentate anterior end of the lower jaw, at least a moderately high coronary process, single lateral row of palatine teeth, relatively small orbit, anteriorly positioned parietal foramen, and a narrowing of the parietal between the adductor chambers and development of a sagittal crest. Diagnosed in an exclusive clade with Pleurosauridae based on a triangular skull in the dorsal view, posteriorly tapering maxilla, posteriorly tapering palatine, moderately open interpterygoid vacuity, pterygoid participation in the suborbital fenestra, low angle of the mandibular symphysis, gracile lower jaw, jaw joint positioned dorsal to the maxillary tooth row and an unossified radiale. Although not formally analysed, this clade is also diagnosed by a dorsoventrally compressed and elongate skull, and elongate external nares.

## 4. Description

### 4.1. General

The anterior third of the ossified skeleton, including skull, forelimbs and the first 18 presacral vertebrae and accompanying ribs, is exposed in the dorsal or dorsolateral view (figure 1). Posterior to this point, the skeleton is slightly displaced and rotated approximately 180° so that it is visible largely in the ventral view. Elements of the left hindlimb lie obliquely across the body's median sagittal plane, exposing them in the dorsal view. The caudal vertebrae comprising the posterior three-quarters of the tail are visible in the left lateral view.

### 4.2. Skull

The antorbital region is short, and the circular orbit is small relative to skull length (figure 2). The elongate postorbital region includes expanded upper and lower temporal fenestrae. The upper temporal arcade, however, does not flare laterally beyond the plane established by the orientation of the maxilla. This lateral restriction produces a skull with an overall triangular shape in the dorsoventral view. The unfused premaxilla bears a short nasal process, strong maxillary process and elongate dorsal process. The latter rises steeply over the maxilla to contact the prefrontal, thereby excluding the maxilla from the nasal and the external naris. The premaxilla houses a chisel-like fang, which appears to reflect a fusion of three incisiform teeth. The maxilla includes a prominent ascending process and an elongate posterior process that tapers to a narrow terminus cupped by the jugal. The maxilla supports 13 or 14 acrodont teeth, the anterior six of which are small and represent the hatchling dentition. These teeth vary somewhat in size but not in an alternating pattern (see [5]). Seven larger and more complex teeth are visible posteriorly. The first of these teeth is smaller than the remaining six. The post-hatchling teeth each include a low posterolingual flange extending from a primary cusp that lacks longitudinal fluting.

Elongate nasals form the medial and posterior margins of the moderately elongate external nares. The finely tapered posterior end of each nasal is wedged between the prefrontal and the frontal. The unsculptured prefrontal forms the entirety of the anterior orbital wall, as there is no lacrimal. The lacrimal duct appears to exit the orbit in the ventral part of the prefrontal, much as in *Sphenodon* (see fig. 2 of [40]). The prefrontal braces the dermal roof and the rostrum against the palate, forming ventral contacts with the palatine and palatal process of the maxilla. The triangular frontals are unfused elements whose greatest width lies along the transverse frontoparietal suture. The frontals contribute laterally to the orbital margin and reach the postfrontal and postorbital despite lacking a distinct posterolateral process. Frontal–postorbital contact negates the small and unsculptured postfrontal from participating in the upper temporal fenestra. The unfused parietals form a broad anterior plate, which is pierced by a teardrop-shaped parietal foramen. The parietals narrow posteriorly to form a sagittal crest. An adductor shelf sweeps ventrally to contact the prootic.

The large postorbital is triradiate. Its anterodorsal process contacts the roofing elements and helps define the rostral end of the upper temporal fenestra. The ventral process forms the posterior orbital margin and runs deep to the jugal. The large caudal process contributes to the upper temporal arcade and defines the anterolateral margin of the upper temporal fenestra. This process shares a long, arching suture with the dorsal process of the jugal. Jugal–squamosal contact negates postorbital participation in the lower temporal fenestra. The jugal lacks even a rudimentary subtemporal process, leaving the lower temporal fenestra completely open ventrally. A short anterior process of the jugal runs over and deep to the maxilla but ends well short of the prefrontal.

The squamosal forms the caudal half of the upper temporal arcade. Its dorsal process contacts the parietal to complete the post-temporal bar (no evidence of a supratemporal). The ventral process follows the convex rostral margin of the quadrate and the quadratojugal, and ends as a finely tapered point just above the jaw joint. The quadratojugal has neither a subtemporal nor a caudal process. It does have an ascending process that reaches, or closely approximates, the vertical midpoint of the temporal plate. The quadrate bears a concave posterior margin that probably supported a tympanic membrane. A sizable quadrate foramen is restricted to the quadrate. There is no visible stapes.

The palate appears largely complete, although it is mostly obscured from the external view. The right and left palatines define the anteromedial margins of an elongate suborbital fenestra. A single and relatively short palatine tooth row is visible in the CT data, although the details of the tooth crowns cannot be discerned. This tooth row is angled oblique to that of the maxilla. Both palatines narrow posteriorly along an angled suture they share with the palatal process of the pterygoid. A long pterygoid–pterygoid contact significantly restricts the rostral terminus of the interpterygoid vacuity. The posterior opening of the vacuity is not markedly wide, but it is also not restricted by medial processes of the pterygoids. The ectopterygoid wing (pterygoid) is set well anterior to the basicranial joint and participates in the suborbital fenestra. There is no evidence of a pterygoid tooth row. The free, rostral end of the cultriform process (parasphenoid) is externally visible dorsal to the pterygoids.

The largely visible left lower jaw is more gracile than that of many deeply nested rhynchocephalians (e.g. *Clevosaurus*, *Sphenodon*). It is more robust than the hyper-elongate jaw of *Pleurosaurus*, which occupies a derived, probably piscivorous morphospace among rhynchocephalians [10]. The shallow horizontal ramus is set well behind the premaxilla, producing a distinctive overbite. Its anterior surface sweeps back at a low angle so that its transition to the ventral margin of the horizontal ramus occurs well behind the anteriormost point of the jaw. This transition is smooth, as the jaw lacks an anteroventral process. The lower dentition is obscured by matrix in the lateral view. The crowns of three dentary teeth, corresponding to the additional (post-successional) dentition [5], are partially visible through the left orbit and left suborbital fenestra. Their morphology includes an elongate rostral flange and a much shorter caudal flange, both extending from a primary cusp. Lateral wear facets are conspicuous, especially on the first two teeth. The CT data are of little help in resolving further details of the tooth cusps; they do demonstrate that the anterior length of the jaw is edentulous. The dentary reaches, but does not extend past, the craniomandibular joint. The joint itself is positioned slightly dorsal to the maxillary tooth row. A large mandibular foramen pierces the elongate dentary–surangular suture. The angular, articular and prearticular all contribute to a pronounced retroarticular process.

### 4.3. Postcranial axial skeleton

The vertebral count is 7 cervicals, 16 dorsals, 2 sacrals and 42 caudals (figures 1 and 3*a–c*). The only exposed centra are those of Dv11 and Dv12 (18th and 19th presacrals), which are amphicoelous. The presence of a notochordal canal can be neither rejected nor confirmed. The neural spines are low, especially in the cervical series, and extend the entire length of the neural arch. Their lateral outline is less triangular than square. The pre- and postzygapophyses are distinctively swollen (figure 3*a*). Secondary articulation surfaces (zygosphene, zygantrum) are difficult to detect in an articulated series [41] but are definitely lacking in Dv11 and Dv12. The cervical ribs at vertebral positions Cv4–6 are holocephalous.

The larger dorsal vertebrae exhibit a successive exaggeration of the zygapophyseal swelling and elongation that begins in the neck (figure 3*a*). The ventral surface of their respective centra is hourglass shaped. There is no evidence of free, presacral intercentra. The neurocentral sutures are fully fused. The holocephalic dorsal ribs are expanded proximally and distally. A large number of disarticulated elements preserved deep to the thoracic region probably represent sternal ribs, uncinate processes and gastralia. Some of these exhibit unusual preservation that we interpret as calcified cartilage. There appear to be remnants of a cartilaginous sternum.

The centra of the two sacral vertebrae are at least partially fused to each other. The second sacral rib bears a strong posterolateral process (figure 3*b*). The caudal series is complete and almost completely articulated. The caudal vertebrae successively decrease in both size and complexity. The anterior caudals bear robust ribs that are dorsoventrally compressed, distally tapering and fully fused to the vertebral body. Haemal arches are present from Cdv2/3 to Cdv28/29. Autotomic septa are present from Cdv11 to Cdv36. It is possible that fracture planes also characterize Cdv3–10, but clear identification at these positions is negated by the haemal arches. In Cdv11–22, the autotomic septa traverse the entire height of the neural arch (figure 3*c*), whereas in Cdv23–36, the dorsal half of the septum is closed. The posterior six caudals lack fracture planes altogether. Ontogenetically, secondary closure of the lepidosaur autotomic septa begins posteriorly and proceeds anteriorly [41–43]. This pattern is congruent with the morphology of *Vadasaurus*. The trajectory in *Vadasaurus* does, however, appear truncated relative to that of *Gephyrosaurus* where the fracture planes of the mid-caudal vertebrae are restricted to the ventral half of the neural arch [43].

### 4.4. Pectoral girdle and forelimb

**Figure 4.**
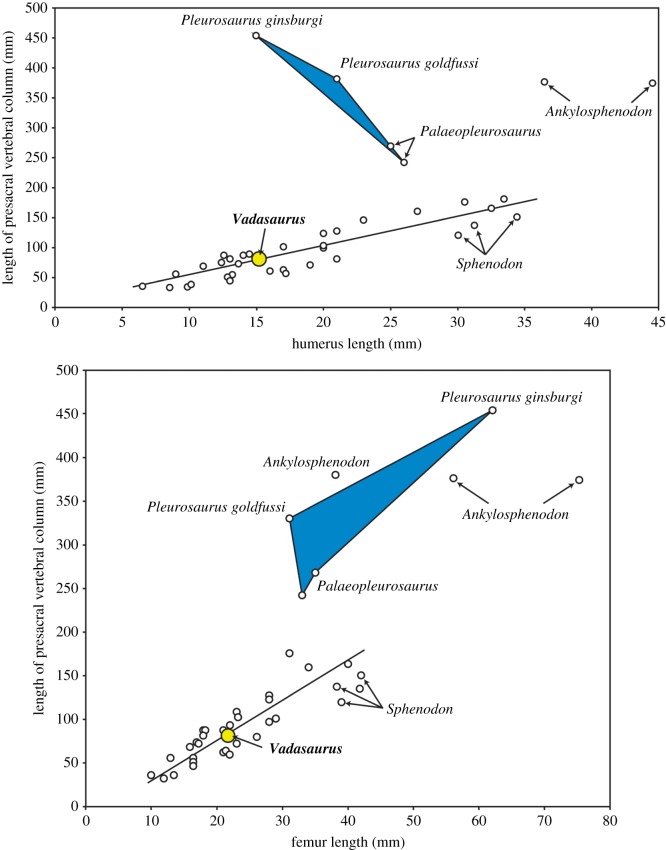
Length relationships between the presacral vertebral column and (*a*) humerus and (*b*) femur. For both indices, *Vadasaurus* conserves a plesiomorphic value, indicating that the derived shortening of the limbs characteristic of Pleurosauridae and especially *Pleurosaurus* spp. is a transformation that postdates the divergence of *Vadasaurus*. Measurements for *Vadasaurus* are from electronic supplementary material, S3. The included lines of best fit do not consider Pleurosauridae, *Ankylosphenodon* or *Vadasaurus*. Comparative data are from Reynoso [44].

The humerus (figures 1 and 3*d*), like the femur (see below), is moderately short but clusters with most other rhynchocephalians when plotted against the length of the presacral vertebral column (figure 4). The humerus is expanded on both ends and its head is capped by a partially fused epiphysis as described above. The radius is robust—comparable in size to the ulna. An ulnar–patellar sesamoid is present (figure 3*d*) (see [45]). The proximal ulnar epiphysis appears only partially fused.

The well-preserved carpals include a large intermedium bearing a distinct notch for a perforating artery. The ulnare is rounded and large*.* An ossified radiale is absent from both wrists. Its presence as a cartilage is indicated by a gap between the radiale and medial half of metacarpal I—a conclusion buoyed by the excellent state of preservation. Based on the size of this gap, the radiale was smaller than the ulnare. A pisiform is not apparent in the left wrist, but may be represented on the right side by a small lateral ossification near the contact of the ulna and the ulnare (figure 3*d*). The lateral (proximal) centrale lies distal to the intermedium and is larger than the medial (distal) centrale. A third central element contacts the intermedium, distal carpals III and IV, and forms a bony bridge between the proximal centrale and the ulnare.

There are four distal carpals but no clear evidence of distal carpal V. The phalangeal formula of the manus is ?-3-4-5-3. Each of the visible unguals bears a prominently curved claw. Digit IV is the longest manual digit, followed successively by digits III, II, V and I. Metacarpal I, and to a slightly lesser degree metacarpal V, are distinctively wide relative to the metacarpals of digits II–IV.

### 4.5. Pelvic girdle and hindlimb

The pelvic bones are disarticulated and slightly displaced from one another (figures 1 and 3*b,e*). The ilium is robust with a strong supra-acetabular buttress. The iliac blade is short and stout with a dorsal ridge and tapered distal terminus. The ilium–pubis contact is reinforced by a slender rostral process of the ilium. The pubis is marked by an expanded and broadly rounded dorsal margin that is pierced by the obturator foramen and is flattened where it accepted the proximal part of the ischium. The pubic contribution to the pubo-ischiatic plate is narrow and rectangular. The arched posterior margin of the pubis indicates a relatively large thyroid fenestra, comparable to other rhynchocephalians. The ischium is slightly larger than the pubis. Its posterior margin includes a large process that would have accepted fibres of the caudal musculature.

The femur exhibits sigmoid flexure and axial rotation with a prominent internal trochanter. The medial and lateral condyles lack distinct separation, and the intertrochanteric fossa is poorly defined. The tibia is only slightly more expanded proximally than distally. The cnemial crest is poorly developed. The fibula is gracile with a modest proximal and distal expansion.

The calcaneum remains unfused to the slightly larger astragalus (figure 3*e*). The pentagonal calcaneum contacts the fibula proximally, astragalus medially and distal tarsals II–IV. The distal end of the astragalus is expanded, with a medially directed boot that reaches the tibia. The astragalus sends a broken, yet sizable, process proximally along the fibular shaft. An obvious free centrale is lacking. The tibia contacts metatarsal I directly, suggesting loss of distal tarsal I. There is no evidence of a metatarsal I–astragalocalcaneal sesamoid [45]. Distal tarsal II is large and contacts metatarsals I and II. The smaller distal tarsal III extends between metatarsals II and III. The small fourth distal tarsal lies between metatarsals III and IV where it lacks broad contacts with the astragalus and metatarsal IV. Instead, these contacts are formed by a rectangular distal element that appears to be at least partially fused to metatarsal V. The identity of this element is unclear but it may represent a second ossification centre in the condensation that plesiomorphically develops as distal tarsal III.

Metatarsal I is short and distinctly robust, with metatarsals II–IV being successively more elongate and gracile (figure 3*e*). Metatarsal V is also broad but approximately half the length of metatarsal I. Its lateral margin bears a hook, but one that is only moderately developed relative to *Sphenodon* (see plate VI of [46]). The pedal phalangeal formula is the expected 2-3-4-5-4. The distal portions of phalanges II–1 and III–1 are reconstructed. The ungual phalanges of the pes, like those of the manus, are mediolaterally compressed and bear grooves on their medial and lateral surfaces (see [47]).

## 5. Phylogenetic analysis

**Figure 5.**
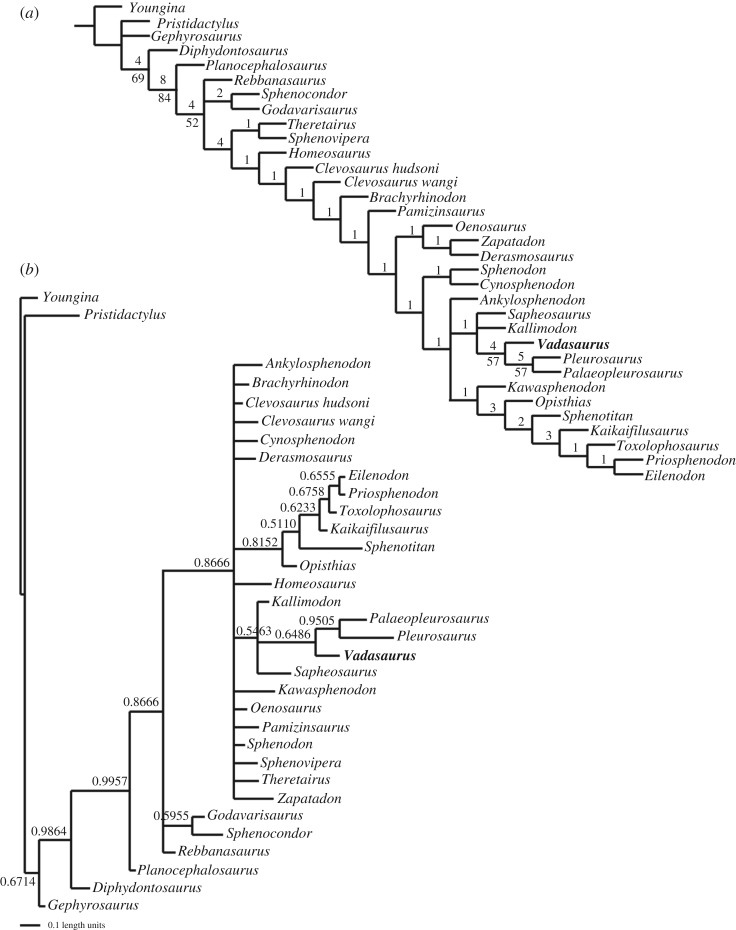
(*a*) Strict consensus of the 12 MPT recovered from the ordered, parsimony analysis. Bremer support and bootstrap values exceeding 50% are provided. TL = 300, CI = 0.440, RI = 0.672. (*b*) Bayesian tree topology derived from the full complement of taxa and with 19 of the 87 characters ordered. Phylogenetic resolution is less than in the parsimony analysis, but the *Vadasaurus*-pleurosaur clade and the endemic European clade are retained with posterior probabilities that exceed the 50% threshold. TL = 355, CI = 0.372, RI = 0.565.

Maximum-parsimony analysis of the taxonomically inclusive matrix with 19 ordered characters produced 12 most parsimonious trees (MPTs). The strict consensus of these trees recovers *Vadasaurus* as the sister taxon to the pleurosaurs (figure 5*a*). This clade is diagnosed by ten synapomorphies, which include: strongly tapered posterior end of the maxilla, reduction of the subtemporal process of the jugal, absence of distinct lateral bowing of the supratemporal arcade, palatines tapering posteriorly and housing a short row of teeth, pterygoid participation in the suborbital fenestra, interpterygoid vacuity with a moderately wide posterior opening, quadrate foramen restricted to the quadrate, gracile lower jaw whose anterior surface in the lateral view sweeps posteriorly at distinctly low angle, jaw joint positioned dorsal to the maxillary tooth row and absence of an ossified radiale.

Alliance of this clade with *Kallimodon* and *Sapheosaurus* indicates a Jurassic radiation of rhynchocephalians endemic to the European Archipelago of the northern Tethys Sea. Evidence for this European clade is not overly strong but does include a pair of synapomorphies—loss of propalinal jaw motion and secondary expansion of a retroarticular process of the jaw. Other characters, such as the relative proximity of the basicranial joint and the lateral process (ectopterygoid wing) of the pterygoid, and expansion of caudal vertebral count to more than 40 (not analysed), may well bolster support for this clade. Unfortunately, these characters are not preserved in phylogenetically important neighbouring taxa (e.g. *Ankylosphenodon*). Statistical support for the clade would increase if *Kallimodon* and *Sapheosaurus* were considered taxonomic synonyms (following [48]; see [17]), and the characters for which they currently express conflicting states, such as a ventral process at the anterior end of the dentary, were scored as polymorphic.

The European clade, in turn, has an exclusive, but unresolved, relationship with *Ankylosphenodon* and Eilenodontidae. The lone extant rhynchocephalian, *Sphenodon punctatus*, lies just outside this group where it is the sister taxon of the middle Jurassic *Cynosphenodon*. The other form we analysed from the Jurassic Plattenkalk of Bavaria is *Homeosaurus.* It is one of the most common fossil sphenodontian taxa and apparently diverged much earlier from the main rhynchocephalian lineage. As expected, the base of the tree is populated by the successively crown-ward divergences of *Gephyrosaurus*, *Diphydontosaurus* and *Planocephalosaurus*.

Analysing all characters as unordered reduces phylogenetic resolution (electronic supplementary material, S2). The *Vadasaurus*-pleurosaur clade is retained, as is most of the resolution within Eilenodontidae (*Kawasphenodon* loses its clear affinity with this radiation). *Gephyrosaurus* and *Diphydontosaurus* retain their basal positions, but the remaining taxa fall into one of two nested polytomies. This diminished resolution is largely the product of converting a single character, relative length of the antorbital region of the skull, to an ordered status. When this character alone is analysed additively, the previous resolution is not only recovered but actually improved upon—producing six rather than 12 MPTs, with *Kallimodon* and *Sapheosaurus* forming the successive sister groups to the AMNH *Vadasaurus*-pleurosaur clade. Removing the nine less-complete taxa did not significantly change the topology.

Of the 26 clades recovered in our ordered analysis, 16 (61.5%) have the minimum Bremer value of 1. This includes the endemic European clade of *Sapheosaurus*-*Kallimodon*-*Vadasaurus*-Pleurosauridae. The *Vadasaurus*-pleurosaur grouping and a monophyletic Pleurosauridae (*Palaeopleurosaurus* + *Pleurosaurus*), however, are actually two of the best supported clades within all Rhynchocephalia with Bremer values of 4 and 5, respectively. Bootstrap support is only 57% and 59% for the two clades; but again, these are two of the higher values in the analysis. The overall weakly supported nature of current parsimony-derived hypotheses for Rhynchocephalia is reinforced by Bayesian inference where posterior probabilities for nearly all the recognized clades dip below the 50% threshold (figure 5*b*). The posterior probability of an exclusive *Vadasaurus*-pleurosaur clade, although still low by general standards, is retained as one of the more probable groupings in the analysis.

## 6. Discussion

**Figure 6.**
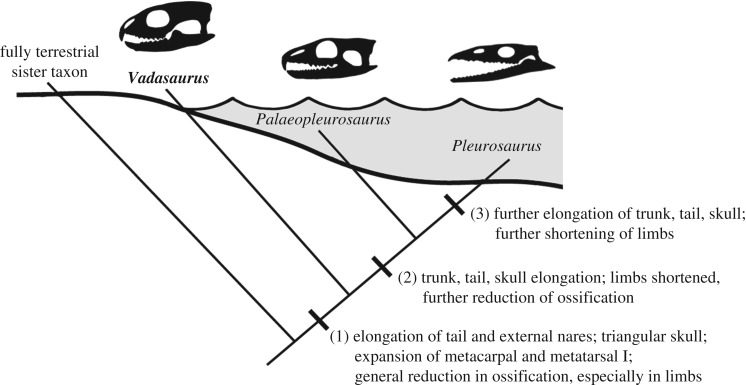
Inferred sequence of selected morphological transformations between the fully terrestrial pleurosaurian ancestor and the marine-adapted *Pleurosaurus*. *Vadasaurus* exhibits the earliest features of this transformation series, many of which are less-exaggerated versions of the derived morphologies expressed in *Palaeopleurosaurus* and especially *Pleurosaurus*. The image depicts the relative degree of aquatic adaptation, not necessarily a literal reconstruction of each taxon's specific ecology.

Recovery of *Vadasaurus* as the sister taxon to Pleurosauridae establishes it as a valuable window into the evolutionary origins of the pleurosaurs and their ecological transition from land to water (figures 5 and 6). Its mosaic of plesiomorphic and derived features supports a more detailed model of character acquisition along the pleurosaur lineage than previously possible and facilitates the articulation of new hypotheses for how the morphological and inferred ecological transformations of this group relate to each other phylogenetically. A series of apomorphies can now be identified as the early steps in the construction of the pleurosaur body plan. These include: (i) expansion of the first metacarpal and metatarsal; (ii) reduction of the prominently hooked fifth metatarsal; (iii) reduced ossification of the epiphyseal joint surfaces; (iv) reduced ossification of the carpals and tarsals, including loss of an ossified radiale; (v) retention of an unfused astragalus and calcaneum; (vi) retention of an unfused scapula and coracoid; (vii) elongation of the tail (caudal vertebral series); (viii) elongation of the external nares; (ix) dorsally positioned jaw joint; and (x) increasingly gracile lower jaw with an edentulous anterior end and low-angled rostral margin. Characters i–v represent the complete list of skeletal features identified as characteristic of secondarily aquatic vertebrates [49]. In turn, characters vi–x were all considered morphological responses of pleurosaurs to their aquatic ecology [16,20]. This includes the evolution of the quick, snapping bite used by many aquatic vertebrates (characters ix and x) [7,50].

The collective expression of these features in *Vadasaurus* constitutes a compelling argument that this taxon, if not fully aquatic, was spending considerable time in the water. The case is strengthened further by the observation that several partially aquatic, extant lepidosaurs lack obvious (i.e. external, macroskeletal) skeletal adaptations for swimming. This includes the Galapagos marine iguana *Amblyrhynchus cristatus*, which feeds exclusively in the intertidal and subtidal zones [51–53]. The absence in *Vadasaurus* of other pleurosaur apomorphies, such as an elongated presacral vertebral column, unfused neurocentral sutures, reduced limb lengths and expanded rostrum, establish these as later transformations in a body plan becoming increasingly streamlined and efficient at both swimming and aquatic feeding.

Histology should help refine our understanding of apomorphy acquisition in the pleurosaur body plan. The ribs and gastralia, but not the femur, of *Palaeopleurosaurus* exhibit a degree of osteosclerosis (increased bone mass) relative to those of terrestrial tetrapods including *Sphenodon* [54]. It will be interesting whether this potential source of ballast [55,56] was in place by the time *Vadasaurus* diverged from the backbone of the pleurosaur line and whether these histological signatures were significantly expanded in *Pleurosaurus*. Independent data such as carbon isotopes [57] may well prove useful in detailing the relationship between ecology and apomorphy accrual.

The use of such ontogenetically dependent characters as ossification levels to help establish phylogenetic patterns raises the question of whether AMNH FARB 32768 is adequately mature in its skeletal development to allow meaningful comparisons with other rhynchocephalian taxa. The complete fusion of all visible neural and neurocentral sutures provides some sense of a lower ontogenetic boundary for the specimen but is not reliable evidence of general skeletal maturity in lepidosaurs [58]. The relatively small orbit is perhaps better evidence. Juvenile vertebrates exhibit larger orbits as a product of precocial brain development (specifically the diencephalon) and its surrounding connective tissue relative to the facial skeleton [59]. This large size is generally reduced over ontogeny as the facial and postorbital regions of the skull expand, but it can be conserved quite late in reptile postnatal development [60–62]. Detailed coefficients of allometry have yet to be established for the rhynchocephalian orbit, but the orbits of juvenile *Sphenodon* certainly exhibit the expected size distinction [7,46,63]. There is a phylogenetically informative decrease in orbit size (relative to total skull length) within Rhynchocephalia [7], which may complicate how the feature informs maturity assessments. However, the transformation itself conforms to a hypermorphic pattern of developmental evolution [64], which only increases the probability that AMNH FARB 32768 is skeletally mature.

The most convincing evidence that AMNH FARB 37268 can be considered skeletally mature is the full fusion of most limb epiphyses to their corresponding long bones. Partially fused epiphyses are probably present at the proximal end of the humerus and ulna, but the remaining elements lack visible epiphyseal plates—this is especially clear in the hindlimb. Fully fused epiphyses are strong indicators of sexual maturity in squamates, although their presence does not necessarily mean that somatic growth has stopped [58]. The lack of complete fusion in the proximal epiphyses of the humerus and ulna may reflect a normal developmental sequence that AMNH FARB 32768 had yet to complete (epiphyses are known to begin calcifying in the hindlimb prior to the forelimb in at least some squamates [58]), or it may represent the initial step in a derived transformation that left the epiphyses of *Vadasaurus* and the pleurosaurs unfused [20]. The latter hypothesis is supported by the suite of other features that correlate with an aquatic ecology (see above).

The presence of fused epiphyses is evidence that the unfused nature of the astragalus–calcaneum and the scapula–coracoid in AMNH FARB 37268 is the product of derived ontogenetic patterning, as these elements typically fuse prior to the epiphyses in lepidosaurs [39,58,65]. An unfused astragalus–calcaneum is a deeply nested synapomorphy shared between *Vadasaurus*, pleurosaurs, *Kallimodon* (but not *Sapheosaurus*), *Ankylosphenodon* and *Sphenodon*. The condition is also present in *Planocephalosaurus*, but here its expression appears to reflect conservation of an earlier transformation; fusion of these elements prior to sexual maturity is considered a lepidosaur synapomorphy [39]. Absence of scapula–coracoid co-ossification is more taxonomically restricted among rhynchocephalians. It is a derived feature shared uniquely among *Vadasaurus*, pleurosaurs, *Sapheosaurus* (but not *Kallimodon*) and *Ankylosphenodon*, and it may also reflect the influence of an aquatic ecology.

The observation that so many anatomically disparate features in these taxa conform to a paedomorphic pattern of evolutionary change [64] raises the question of mechanism. As with *Vadasaurus*, it is difficult to infer how much time, if any, *Sapheosaurus* and *Kallimodon* were spending in the water [20,44,66] and thus what role an aquatic ecology might have played in the retention of the unfused states they share with pleurosaurs. It is conceivable that a move to the water eased the selective pressures that otherwise constrain the later stages of skeletal development and that are probably responsible for such widespread conservation of these co-ossifications. One might predict that a lessening of such constraints would increase variability in the later stages of skeleton formation and thus in the expression of these co-ossifications (see [67]). The proximal regulation of these paedomorphic trajectories may well have been autocrine in nature, which is congruent with the early conceptualization of heterochrony as an evolutionary process that affects numerous features simultaneously [68]. The fact that not all the expected elements in these taxa fail to co-ossify (e.g. the long-bone epiphyses and neurocentral sutures) may reflect complexity in the downstream regulation of the involved autocrine signal(s), including the role of functional constraints. Certainly, until we better understand the underlying mechanisms and hierarchical relationships of skeletal heterochrony, the mosaic nature of these expression patterns warns against analysing the various hypo-ossified states in rhynchocephalians as a single character. With this in mind, we adopted the premise that these skeletal signatures are developing and evolving as independent character systems.

*Ankylosphenodon* from the Albian of central Mexico is the only other rhynchocephalian considered to be non-obligate aquatic, and it too exhibits reduced ossification in several features [44]. We might presume this ecology is convergent, given the stratigraphic and geographical disparity between it and the European forms, although we should be wary of permitting geography and stratigraphy to influence hypotheses of taxonomy and homology [69,70]. Removing from the analysis those synapomorphies most probably associated with an aquatic habitus does result in *Ankylosphenodon* moving to the base of Eilenodontidae. At this position, its shearing bite and inferred herbivory are homologous with the other taxa of that group [71]. Even under this topology, the possibility remains that rhynchocephalians became semi-aquatic only once with both the marine pleurosaurs and the terrestrial eilenodontids evolving out of this ancestral ecology.

## 7. Conclusion

As a lineage is sampled successively closer to its evolutionary origin, one can expect the number of apomorphies diagnosing that lineage to decrease. This logical product of the evolutionary model renders the basal-most taxa in a particular transformation sequence, and thus the tempo and mode of character evolution in the earliest history of that sequence, difficult to reconstruct with generally acceptable levels of statistical support. Given this point, the evidence is perhaps surprisingly strong that *Vadasaurus* from the Late Jurassic marine limestones of Solnhofen is the sister taxon to Pleurosauridae and constitutes a morphological window into the early history of a transformation sequence that produced the fully marine *Pleurosaurus*. The initial apomorphies of that sequence are distributed throughout the skeleton and include reductions in ossification/fusion as well as transformations that more obviously increase hydrodynamic efficiency for both swimming and aquatic feeding. There is compelling evidence that the lineage entered the water prior to the common ancestor of *Vadasaurus* and Pleurosauridae and possibly before the common ancestor of *Kallimodon/Sapheosaurus*, *Vadasaurus* and Pleurosauridae.

The timing and tempo of morphological change and lineage divergence in the deep history of pleurosaurs remains poorly constrained. The early Jurassic (Toarcian) age of *Palaeopleurosaurus* sets a hard minimum for these events, but the lineage as a whole must be considerably older given the Triassic age for *Sphenotitan* [71], which is nested in the closely related Eilenodontidae. This minimum age coincides closely with the initial break-up of Pangea and the associated opening of the Tethys Sea that separated Laurasia and Gondwana [72]. The *Kallimodon*/*Sapheosaurus*-pleurosaur clade was thus in place and achieved taxonomic diversity during a time when the northern margin of the Tethys was expanding as an epicontinental sea that filled the various basins of what is now southern Germany and southeastern France [73].

Whereas the support values for a *Vadasaurus*-pleurosaur clade are relatively strong, the topology of the deeply nested lineages within Rhynchocephalia remains unstable. This includes the detailed relationships of the modern tuatara, *Sphenodon punctatus*. The weak support values for most of the recovered clades are not necessarily unexpected given the low ratio of characters-to-terminals and the high degree of character conflict present in the matrix. What remains unclear is to what degree this volatility is a product of inadequate character sampling—i.e. we have yet to identify a significant number of phylogenetically informative features that do exist—versus a true reflection of evolutionary rates within Rhynchocephalia. Testing this question will require using advanced imaging technology to re-examine existing specimens, especially the details of such poorly sampled regions as the palate and braincase, as well as the adequate description and new collection of additional specimens and taxa. The morphological details examined in this study and the questions those details raise suggests that rhynchocephalians still have much to teach us regarding the interplay between development, morphology, ecology and macroevolutionary patterns of taxonomic diversity.

## Supplementary Material

Supplementary Materials

## Supplementary Material

Nexus File of Phylogenetic Matrix

## Data accessibility

Electronic supplementary material, S1–S6, are included as supplementary files at *Royal Society Open Science*.
