# Supplementary material for: A new rhynchocephalian (Reptilia: Lepidosauria) from the Late Jurassic of Solnhofen (Germany) and the origin of the marine Pleurosauridae
Source: R Soc Open Sci. 2017 Nov 8;4(11):170570. doi: 10.1098/rsos.170570 (PMC5717629; doi:10.1098/rsos.170570)
Supplement: Supplementary Materials [file rsos170570supp1.docx]

Electronic supplementary material for “**A New Rhynchocephalian (Reptilia: Lepidosauria) from the Upper Jurassic of Solnhofen (Bavaria) and the Origin of the Marine Pleurosauridae”**

GABRIEL S. BEVER^1, 2*^ and MARK A. NORELL^2^

^1^Center for Functional Anatomy and Evolution, Johns Hopkins University School of Medicine, Baltimore, MD. [gbever1@jhmi.edu](mailto:gbever1@jhmi.edu%22mailto:gsbever@nyit.ed)

^2^Division of Paleontology, American Museum of Natural History, Central Park West at 79th Street, New York, NY 10024. [norell@amnh.org](mailto:norell@amnh.org)

*corresponding author

**Contents**

**S1.** Character List

**S2.** Supplementary Figure

**S3.** Supplementary Table

**S4.** Supplementary References

**S1.** **List and Description of Characters Used in the Phylogenetic Analyses.** All but the last three characters were taken from previous studies. Most correspond to the character list of Apesteguía et al. (2012), which cites the bibliographic use of each character. Those not from Apesteguía et al. (2012) or that have been modified from that study are individually cited. Characters 1, 21, 24, 32, 39, 41, 42, 45, 49, 50, 52, 54, 58, 60, 61, 62, 63, and 64 were identified as morphoclines following Cau et al. 2014. To this list we added character 87. These 19 characters were considered as non-additive and additive in separate iterations of our analysis.

- 1. Antorbital region, length relative to skull length: (0) 1/3 or more; (1) btwn 1/4 and 1/3; (2) 1/4 or less.
  2. Orbit diameter/skull length ratio: Orbit diameter/skull length ratio: (0) 1/3 or greater; (1) less than 1/3.
  3. Supratemporal fenestra/orbit length ratio: (0) 75% or less; (1) more than 75%.
  4. Supratemporal fenestra/skull length ratio: (0) 1/4 or less; (1) more than ¼.
  5. Lower temporal fenestra/skull length ratio: (0) 1/4 or less; (1) more than ¼.
  6. Maxilla, premaxillary process: (0) elongate; (1) short or absent.
  7. Premaxilla, maxillary process that excludes maxilla from external naris: (0) absent; (1) present.
  8. Maxilla, shape of posterior end: (0) tapering posteriorly or very narrow; (1) dorsoventrally broad.
  9. Lacrimal: (0) present; (1) absent.
  10. Jugal, shape of dorsal process: (0) broad and short; (1) narrow and elongate.
  11. Prefrontal and postfrontal, sculpturing: (0) absent; (1) present.
  12. Prefrontal-jugal contact: (0) absent; (1) present.
  13. Postorbital, marked dorsal ridge and deep ventrolateral concavity: (0) absent; (1) present.
  14. Frontals, relation: (0) separated; (1) fused.
  15. Parietals, relation: (0) separated; (1) fused.
  16. Parietal, width between supratemporal passages relative to interorbital width: (0) broader; (1) narrower.
  17. Parietal crest: (0) absent; (1) present.
  18. Parietal, shape of posterior edge: (0)* greatly incurved inward; (1) slightly incurved inward; (2) convex.
  19. Parietal foramen, position relative to anterior border of supratemporal fenestra: (0) posterior; (1) at the same level or anterior.
  20. Lower temporal bar, shape/position: (0) aligned with maxillary toothrow; (1) bowed away beyond limit of adductor chamber.
  21. Lower temporal bar, subtemporal (posteroventral) process of jugal: (0) absent; (1) poorly to moderately developed <1/2 length of lower temporal fenestra; (2) well developed 1/2 length of lower temporal fenestra or more.
  22. Palatine, shape of posterior end: (0) tapers posteriorly; (1) widens posteriorly.
  23. Pterygoids, anterior contact (modified by Rauhut et al. 2012, 25): (0) absent; (1) slight; (2) vast.
  24. Pterygoids, posterior opening of interpterygoid vacuity between posteromedial processes: (0) widely open; (1) moderately open, as wide as vacuity; (2) almost closed by posteromedial processes.
  25. Pterygoid, posterior border of ectopterygoid wing (modified by Rauhut et al., 2012, 27): (0) approximately level with basicranial articulation; (1) offset anteriorly from basicranial articulation.
  26. Pterygoid, participation in margin of suborbital fenestra: (0) forms part of margin; (1) excluded.
  27. Quadrate-quadratojugal foramen, relative size: (0) small; (1) large.
  28. Quadrate-quadratojugal foramen, position: (0) between quadrate and quadratojugal; (1) entirely within quadrate.
  29. Quadrate-quadratojugal, conch size and emargination: (0) pronounced; (1) reduced.
  30. Suparatemporal, discrete bone: present (0); fused or absent (1).
  31. Inferred jaw motion: orthal (0); propalineal (1).
  32. Degree of propalinality, measured either as palatal toothrow extension or length over which palatines keep parallel to maxillae: (0) small palatal row, parallel line restricted to anterior region; (1) enlarged, palatines accompanying maxilla for 1/2 its own length; (2) palatines accompany maxilla for complete.
  33. Mandibular symphysis, mentonian process: (0) reduced or absent; (1) well developed and pointed; (2) well developed and rounded.
  34. Mandibular symphysis, shape: (0) almost circular, height-length relation near 1; (1) oval, height/length clearly greater than 1.
  35. Lower jaw, anterior region: (0) toothed; (1) edentate.
  36. Dentary, ventral process at anterior end: (0) absent; (1) well developed.
  37. Inclination of jaw symphysis in relation to long axis of the mandible (line from anterodorsal end of dentary to posterior end of glenoid (modified by Rauhut et al., 2012, 38): (0) more than 50 deg.; (1) less than 50 deg.; (2) almost vertical ventrally with pronounced anterior process dorsally.
  38. Mandibular symphysis, dorsal development (symphyseal spur) (Rauhut et al., 2012, 39): moderately developed (0); well projected, comparable to a caniniform (1).
  39. Mandibular foramen, size: small (0); large (1).
  40. Glenoid cavity, shape: (0) smooth surface, lacking anteroposterior central ridge; (1) elongate and asymmetrical surface, with a strong anteroposterior central ridge; (2) symmetrical facet with strong anteroposterior central ridge.
  41. Coronoid process, height relative to that of the jaw at the level of the anterior end of the coronoid process: (0) low, weak, less than 1/2 jaw; (1) moderately high, around 1/2 jaw height; (2) very high, nearly as high as jaw.
  42. Retroarticular process, shape: (0) pronounced; (1) reduced, caudally projected; (2) reduced, dorsally curved.
  43. Dentary, posterior process, relative length: (0) short, not reaching glenoid level; (1) elongate, reaching glenoid level; (2) elongate, reaching end of glenoid level.
  44. Marginal dental implantation, type: (0) pleurodont; (1) degree of posterior acrodonty; (2) fully acrodont.
  45. Tooth replacement, type: (0) alternate; (1) addition at back of jaw.
  46. Dentary regionalization with small juvenile teeth (hatchling) in anterior region of maxilla and dentary: (0) absent, only pleurodont teeth; (1) present, with hatchling pleurodont teeth; (2) present, with hatchling, succesional and additional acrodont teeth; (3) absent in both juveniles and adults, only additional acrodont teeth.
  47. Dentary, posterior successionals, number in mature individuals (Apesteguía et al. 2012, 46): (0) zero; (1) one; (2) two or more.
  48. Marginal teeth, lateral wear facets on dentary and/or medial wear facets on maxilla: (0) absent or smooth; (1) present, conspicuous.
  49. Marginal teeth, shape of cross-section of posterior teeth: (0) breadth equal to or less than length; (1) square, expanded lateromedially; (2) rectangular, wider than long.
  50. Premaxillary teeth, number in mature individuals: (0) more than 7; (1) 7 to 4; (2) 3 or fewer.
  51. Premaxillary teeth, morphology: (0) present as discrete teeth; (1) merged into chisel-like structure.
  52. Maxillary teeth, posteromedial flanges on posterior teeth: (0) absent, simple cone; (1) present as small flanges on at least one tooth; (2) present as extensive flanges on most teeth.
  53. Maxillary teeth, anterolateral flange on posterior teeth: (0) absent; (1) present.
  54. Palatine teeth, number of tooth rows: more than one (0); a single row plus one isolated relictual tooth (1); single lateral tooth row (2). (0) 2 or more; (1) single row + one isolated tooth; (2) single lateral row.
  55. Palatine teeth, flanges: (0) completely absent; (1) present, on at least some teeth.
  56. Pterygoid teeth, number of tooth rows: (0) 3 or more; (1) two; (2) one or none.
  57. Mandibular teeth, anterolateral flanges: (0) absent; (1) present, at least in 1 tooth.
  58. Mandibular teeth, anteromedial flanges: (0) absent; (1) present.
  59. Dental ridges in adult additional teeth: (0) absent; (1) present.
  60. Second sacral vertebrae: (0) present, small; (1) present, prominent; (2) absent.
  61. Second sacral rib, bifurcated: (0) absent; (1) present.
  62. Ischium, process on posterior border: (0) absent, posterior end uninterrrupted; (1) present as small tubercle; (2) present as prominent process.
  63. Skull, length (as an estimation of the body size): (0) small, less than 3 cm; (1) medium, btwn 3 - 10 cms; (2) larger > 10cm.
  64. Dentary, proportions (length/height ratio): (0) gracile, long and low, < 0.18; (1) average, between 0.18 and 0.28; (2) robust short and high, > 0.28.
  65. Dentary, successional teeth, max concurrent number of ontogeny: (0) 6 or more; (1) 3 to 5; (2) 2 or fewer.
  66. Dentary, anterior successional teeth (not "caniniform"), number in adult: (0) 2 or more discrete teeth; (1) 1 or 2 poorly distinct ; (2) none or indistinct.
  67. Dentary, successional teeth, striations: (0) present; (1) absent.
  68. Dentary, posterior successional teeth, lingual groove: (0) absent; (1) present.
  69. Dentary, hatchling teeth, striations: (0) absent; (1) present.
  70. Dentary, successional "caniniform", shape of basal cross section: (0) nearly circular; (1) clearly oval, labially-lingually compressed.
  71. Metatarsal V shape (Cau et al. 2014, 74): (0) straight; (1) hooked.
  72. Postzygapophyses, dorsal shape (modified from Cau et al. 2014, 75): (0) flat; (1) swollen.
  73. Scapulocoracoid suture (Cau et al. 2014, 76): (0) fused; (1) open.
  74. Astragalus and calcaneum (Cau et al. 2014, 77): (0) unfused ; (1) fused
  75. Metatarsal V, length relative to other metatarsals (Cau et al. 2014, 78): much shorter (0); subequal (1).
  76. Posterior dorsal centra and ribs (Cau et al. 2014, 79): short centra with long ribs (0); elongate centra with short ribs (1).
  77. Metapodials, general shape (Cau et al. 2014, 80): gracile and slender, bones spreading distally (0); stout and robust, bones appressed (1).
  78. Palatines, midline contact (Rauhut et al. 2012, 24): (0) absent, separated by pterygoids/vomer; (1) present, no pterygoid/vomer contact.
  79. Parabasisphenoid, depression (Dupret, 2004, 27): (0) absent; (1) present
  80. Anterior caniniform teeth in maxillae and dentaries (Dupret 2004, 31): (0) absent; (1) present.
  81. Lateral palatine tooth row (Rauhut et al. 2012, 57): curves medially posteriorly (0); parallel to maxillary tooth row over entire length (1).
  82. Presacral vertebrae, number (Rauhut et al. 2012, 63):(0) less than 25; (1) more than 25
  83. Maxillary teeth, relative size of posterior and anterior teeth (modified by Rauhut et al. 2012, 53): (0) subequal; (1) posterior teeth smaller.
  84. Premaxilla, nasal process length (new): (0) short; (1) long.
  85. Radiale (new): (0) ossified; (1) unossified or absent.
  86. Metacarpal I, breadth relative to other metacarpals (new): (0) subequal, (1) much wider.
  87. Metacarpal I, length relative to that of MC II (new): (0) >80%, (1) 60 - 80%, (2) <60%.


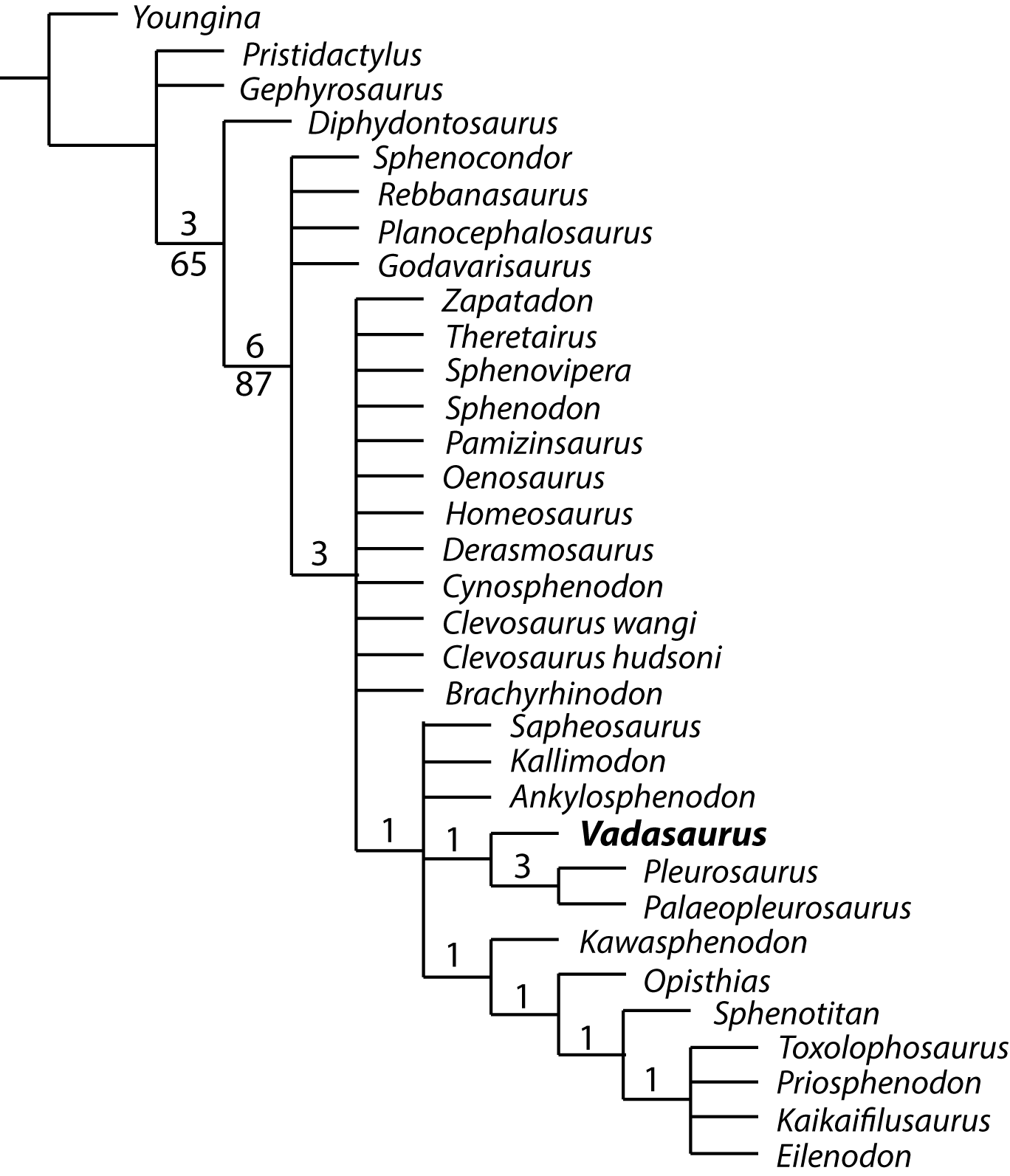
**S2. Supplementary Figure.** Strict consensus of 812 MPT recovered from an analysis in which all characters were analyzed as unordered. The result is a considerable loss in overall phylogenetic resolution, although the *Vadasaurus*-pleurosaur clade is retained. Bremer support and bootstrap values exceeding 50% are provided. TL = 373, CI = 0.354, RI = 0.529.

**S3. Supplementary Table.** **Selected measurements of AMNH FARB 37268 (in mm).** CVL = Length of tail (caudal vertebrae). PSVL = Presacral length of the vertebral column. (L) = left, (R) = right.

| Skull Length | Mandible Length | PSVL | CVL | Humeral Length | Femoral Length |
| --- | --- | --- | --- | --- | --- |
| 31.1 | 29.2 | 81.0 | 160 | 15.3 (R), 15.8 (L) | 21.6 (R), 22.4 (L) |

**S4. Supplementary References**

Apesteguía S, Gómez RO, Rougier GW. 2012. A basal sphenodontian (Lepidosauria) from the Jurassic of Patagonia: new insights on the phylogeny and biogeography of Gondwanan rhynchocephalians. *Zool. J. Linn. Soc.* **166**, 342-360. (doi:10.1111/j.1096-3642.2012.00837)

Cau A, Baiano MA, Raia P. 2014. A new sphenodontian (Reptilia, Lepidosauria) from the Lower Cretaceous of southern Italy and the phylogenetic affinities of the Pietraroia Plattenkalk rhynchocephalians. *Cretaceous Res.* **49**, 172-180. (doi:10.1016/j.cretres.2014.02.001)

Dupret V. 2004. The pleurosaurs: anatomy and phylogeny. *Rev. Paléobiol, Genève. Vol. spéc.* **9**, 61-80.

Rauhut OWM, Heyng AM, López-Arbarello A, Hecker A. 2012. A new rhynchocephalian from the Late Jurassic of Germany with a dentition that is unique amongst tetrapods. *PLoS ONE* **7**, e46839. (doi:10.1371/journal.pone.0046839)

Reynoso V-H. 2000. An unusual aquatic sphenodontian (Reptilia: Diapsida) from the Tlayua Formation (Albian), central Mexico. *J. Paleontol.* **74**, 133-148. (doi:10.1666/0022-3360(2000)074<0133:AUASRD>2.0CO;2)
